# Predictors and Outcomes of Cardiac Events following Thoracic Endovascular Aortic Repair in Descending Thoracic Aortic Aneurysm and Dissection

**DOI:** 10.1055/s-0040-1701606

**Published:** 2020-06-29

**Authors:** Derrick O. Acheampong, Philip Paul, Percy Boateng, I. Michael Leitman

**Affiliations:** 1 Department of Surgery, Icahn School of Medicine at Mount Sinai, New York, New York

**Keywords:** cardiac events, TEVAR, aortic aneurysm, risk factors, endovascular, ACS-NSQIP

## Abstract

**Background** Cardiac events following thoracic endovascular aortic repair (TEVAR) have been associated with significant morbidity and mortality. However, predictors of post-TEVAR cardiac events in descending thoracic aortic aneurysm or dissection are poorly understood.

**Methods** A retrospective analysis of completed TEVAR procedures performed from 2010 to 2016 was conducted using the ACS-NSQIP (American College of Surgeons National Surgical Quality Improvement Program) participant user file database. Adult patients (≥18 years) who underwent TEVAR for descending thoracic aortic aneurysm or dissection were identified and 30-day outcomes were examined. An initial univariate analysis was performed to determine associations between all patient variables and cardiac events, defined as myocardial infarction or cardiac arrest that occurred ≤30 days of surgery. Multivariate logistic regression was subsequently performed to identify independent risk factors for cardiac events following TEVAR.

**Results** The study identified 150 out of 2,905 (5.2%) patients who underwent TEVAR for descending thoracic aortic aneurysm or dissection who developed cardiac events. No significant difference in incidence of cardiac events was noted among patients presenting with aortic aneurysm or dissection ( *p* = 0.339). The overall 30-day mortality rate for all patients was 9.1%. Independent preoperative predictors of post-TEVAR cardiac events included emergency procedure (odds ratio [OR] 2.80, 95% confidence interval [CI] 1.9–4.1, *p* < 0.01); American Society of Anesthesiologists score >3 (OR 1.71, 95% CI 1.1–2.6, *p* = 0.01), ventilator dependence (OR 2.33, 95% CI 1.3–4.2, *p* < 0.01), renal failure (OR 2.53, 95% CI 1.50–4.3, *p* < 0.01), blood transfusion (OR 1.84, 95% CI 1.1–3.2, *p* = 0.03), and preoperative leukocytosis (OR 2.45, 1.6–3.8, *p* < 0.01). After TEVAR, unplanned reintubation (OR 5.52, 95% CI 3.5–8.8, *p* < 0.01), prolonged mechanical ventilation (OR 1.94, 95% CI 1.2–3.2, *p* = 0.011), and postoperative blood transfusion (OR 4.02, 95% CI 2.70–6.0, *p* < 0.01) were independent predictors of cardiac events. Cardiac events greatly increased mortality (60.7 vs. 5.5%), total length of hospital stay (13.2 ± 14.7 days vs. 8.3 ± 9.3 days), and readmission rates (19.3 vs. 8.2%, *p* < 0.01).

**Conclusions** Cardiac events following TEVAR are associated with significant mortality. Patients with these risk factors should be appropriately monitored to improve outcomes.

## Introduction

Thoracic endovascular aortic repair (TEVAR) is becoming a preferable approach for treating thoracic aortic pathologies due to its reduced perioperative morbidity and mortality. Nonetheless, major postoperative complications have been reported. This study identified risk factors for cardiac events (CEs), defined as either myocardial infarction (MI) or cardiac arrest ≤30 days following TEVAR in descending thoracic aortic aneurysm or dissection. Understanding these risk factors should allow for appropriate resources to be given to at-risk patients to improve morbidity and mortality following TEVAR.

Cardiac events continue to remain a significant cause of morbidity and mortality following TEVAR. 1 2 This makes it necessary to determine risk prediction models that would allow surgeons to identify at-risk patients to provide appropriate perioperative management that would minimize morbidity and improve post-TEVAR outcomes. The present study aims to identify risk factors for post-TEVAR CEs in descending thoracic aortic aneurysm or dissection to allow for optimal management of at-risk patients. It is our hope that by providing a practical risk assessment model, surgeons will have a better insight and understanding of at-risk patients to allow for more targeted perioperative management that might lead to improvement of outcomes.

## Materials and Methods

### Data Source and Acquisition

Data were retrospectively extracted from the ACS-NSQIP (American College of Surgeons National Surgical Quality Improvement Program) database from 2010 to 2016. The ACS-NSQIP is a large risk-adjusted database that was originally created in 1994 to provide 30-day surgical outcomes from the Veteran Affairs Healthcare Systems. 3 Currently, the database comprises over 670 hospitals around the world, with patient data collected by trained nurse reviewers. Overall, the database includes over 150 preoperative and intraoperative variables and 30-day postoperative outcomes.

### Disclaimer

The American College of Surgeons National Surgical Quality Improvement Program and the hospitals participating in the ACS-NSQIP are the source of the data used herein; they have not verified and are not responsible for the statistical validity of the data analysis or the conclusions derived by the authors.

### Inclusion Criteria

Adult patients, 18 years and older, who underwent TEVAR were identified using current procedural terminology (CPT) codes 33880, 33881, 33883, 33886, 33889, 33891. Patients included for analyses met the following criteria: (1) indication for TEVAR was for aortic pathologies of descending thoracic aortic aneurysm or dissection, and (2) postoperative outcome variable for MI and cardiac arrest was documented and not missing. Aortic dissections could not be subgrouped into Stanford Type A or Type B since this information is not provided in the ACS-NSQIP database.

Cardiac arrest was defined as the absence of cardiac rhythm or the presence of chaotic cardiac rhythm that results in loss of consciousness requiring the initiation of any component of basic and/or advanced cardiac life support. Myocardial infarction was defined as a new transmural acute MI occurring during surgery or within 30 days following surgery as manifested by new Q-waves on ECG (electrocardiogram). 4

### Variable Definition

Patient demographics included age, gender, and race (white, black, Asian, other); other race was made up of American Indians, Alaska Native, Native Hawaiian, Pacific Islander, and unknown or not reported. Other preoperative variables analyzed included body mass index, American Society of Anesthesiologists (ASA) score classification, emergency surgery, smoking (within 1 year of surgery), alcohol abuse (at least 2 drinks per day), dyspnea (at rest or with moderate exertion) ≤30 days prior to surgery, wound infection, diabetes (noninsulin dependent or insulin dependent), dependent functional health status (partially or totally dependent) ≤30 days prior to surgery, ventilator dependence ≤48 hours prior to surgery, history of chronic obstructive pulmonary disease ≤30 days prior to surgery, pneumonia, congestive heart failure, MI, angina, previous cardiac surgery or percutaneous coronary intervention, hypertension requiring medications, renal failure (acute renal failure and dialysis), transient ischemic attack, cerebral vascular accident (with or without neurological deficits), hemiplegia or paraplegia, tumor or brain occupying lesions in the central nervous system, disseminated cancer, wound infection, weight loss (≥10% of body weight in last six months), steroid use, bleeding disorder (which includes hypothrombotic conditions such as Vitamin K deficiencies, hemophilia, thrombocytopenia, and long-term anticoagulant use, though aspirin is not included within this categorization), blood transfusion of whole/packed red blood cells (PRBCs) in 72 hours prior to surgery, prior operation within 30 days, and systemic sepsis (systemic inflammatory response syndrome [SIRS], sepsis, and septic shock).

Available preoperative laboratory variables analyzed included serum sodium, blood urea nitrogen (BUN), creatinine, albumin, total bilirubin, aspartate aminotransferase (serum glutamic-oxaloacetic transaminase [SGOT]), alkaline phosphatase, white blood count (WBC), hematocrit, platelet count, prothrombin time, partial thromboplastin time, and international normalized ratio (INR). Intraoperative variables included operative and anesthesia times.

Thirty-day postoperative adverse outcomes as predictors of CEs following TEVAR were also analyzed. These included pneumonia, unplanned reintubation, pulmonary embolism, prolonged mechanical ventilation (>48 hours following surgery), renal complications (progressive renal insufficiency, acute renal failure, urinary tract infection), neurological complications (stroke or cerebral vascular accidents, coma >24 hours, peripheral nerve injury), packed RBC transfusions, deep venous thrombosis (DVT), surgical site infection, wound disruption, systemic sepsis, and unplanned return to the operating room.

The associations between CEs and mortality, readmission, and total length of hospital stay were also analyzed.

### Statistical Analysis

Statistical analyses were performed using SPSS software (Version 22, IBM Corporation, Chicago, IL). Descriptive analysis was reported as mean ± standard deviation for continuous data, and percentages for categorical data. Patients were grouped into those who experienced CEs and those who did not, and the risk factors were analyzed. Univariate analysis was performed using chi-square tests to correlate baseline preoperative, intraoperative, and postoperative risk factors with post-TEVAR CEs. All variables that had a *p* -value of less than 0.05 in the univariate analysis were subsequently included in the final multivariate logistic regression with CEs as the dependent variable.

This study was reviewed and approved by the Icahn School of Medicine at Mount Sinai Institutional Review Board.

## Results

The present study identified a total of 2,905 patients who underwent TEVAR from 2010 to 2016, 150 (5.2%) of whom experienced CEs. The overall 30-day mortality rate for all patients was 9.1%. Patients who experienced post-TEVAR CEs had a significantly high incidence of mortality (60.7 vs. 5.5%, *p* < 0.01) than those who did not. TEVAR was performed more frequently in descending thoracic aortic aneurysm than dissection (64.3 vs. 35.7%). There was no significant difference in the incidence of TEVAR among aortic aneurysm or dissection patients ( *p* = 0.339). Of the patients who experienced CEs post-TEVAR, 67.3% presented with aortic aneurysm compared with 32.7% with dissection.

Table 1 summarizes univariate analysis for patient demographics, comorbidities, and perioperative factors on risks of CEs. ASA >3, emergency surgery, dependent functional status, preoperative ventilator dependence, renal failure, bleeding disorder, blood transfusion, systemic sepsis, BUN ≥23 mg/dL, creatinine ≥1.2, albumin <3.5 mg/dL, total bilirubin >1.2 mg/dL, SGOT >35 U/L, alkaline phosphate >126 U/L, WBC >11 K/uL, hematocrit <34%, INR >1.5, and operative time >180 minutes were significantly associated with CEs following TEVAR.

**Table 1:** Univariate analysis of patient demographics, comorbidities, and perioperative factors on the risk of cardiac events following thoracic endovascular aortic repair

Variables	Cardiac events *N* = 150	% Cardiac events	No Cardiac events *N* = 2,755	% No Cardiac events	*p* -Value
*Demographics:*					
Age in years (mean ± SD)	68.9 ± 12.93		68.1 ± 13.02		0.498
BMI in kg/m ^2^ (mean ± SD)	28.2 ± 6.83		28.7 ± 6.60		0.38
*Gender:*					
Male	79	52.7%	1,584	57.5%	0.248
Female	71	47.3%	1,171	42.5%	
*Race:*					
Black	36	24.0%	499	18.1%	0.290
White	96	64.0%	1,898	68.9%	
Asian	4	2.7%	102	3.7%	
Other/Unknown	14	9.3%	256	9.3%	
BMI > 30 kg/m ^2^	45	30.0%	951	34.5%	0.260
Age > 65 y	99	66.0%	1,810	65.7%	0.932
*Comorbidities:*					
ASA score >3	117	78.0%	1,438	52.2%	< **0.01** a
Emergency procedure	80	53.3%	543	19.7%	< **0.01** a
Tobacco smoking	48	32.0%	876	31.8%	0.966
ETOH	105	0.7%	19	0.7%	0.948
Dyspnea	27	18.0%	504	18.3%	0.919
Dependent functional status	19	12.7%	149	5.4%	< **0.01** a
Diabetes	19	12.7%	347	12.6%	0.980
Ventilator dependence	24	16.0%	69	2.5%	< **0.01** a
COPD	29	19.3	460	16.7	0.394
Pneumonia	0	0%	11	0.4%	0.738
CHF	4	2.7%	77	2.8%	0.906
Myocardial infarction	2	1.3%	13	0.5%	0.362
Previous cardiac surgery or PCI	13	8.7%	212	7.7%	0.752
Angina	3	2.0%	35	1.3%	0.659
Hypertension	126	84.0%	2325	84.4%	0.898
Peripheral vascular disease	5	3.3%	52	1.9%	0.411
Renal failure	22	14.7%	132	4.8%	< **0.01** a
TIA	2	1.3%	41	1.5%	0.941
CVA with or without neurological deficits	2	1.3%	77	2.8%	0.551
Hemiplegia/Paraplegia	3	2.0%	16	0.6%	0.109
Tumor or brain occupying lesion in CNS	0	0%	3	0.1%	0.902
Disseminated cancer	1	0.7%	22	0.8%	0.825
Wound infection	6	4.0%	50	1.8%	0.052
Steroid use	11	7.3%	143	5.2%	0.245
Weight loss	4	2.7%	55	2.0%	0.595
Bleeding disorder	31	20.7%	314	11.4%	< **0.01** a
Blood transfusion	28	18.7%	110	4.0%	< **0.01** a
Systemic sepsis	38	25.3%	231	8.4%	< **0.01** a
Surgery within 30 d of TEVAR	3	2.0%	61	2.2%	0.879
*Preoperative laboratory:*					
Sodium <135 mEg/L	24	16.0%	328	11.9%	0.326
Blood urea nitrogen ≥23 mg/dL	57	38.0%	769	27.9%	**0.012** a
Creatinine ≥1.2 mg/dL	68	45.3%	900	32.7%	< **0.01** a
Albumin <3.5 mg/dL	74	49.3%	858	31.1%	< **0.01** a
Total bilirubin >1.2 mg/dL	14	9.3%	151	5.5%	< **0.01** a
SGOT >35 U/L	22	14.7%	224	8.1%	< **0.01** a
Alkaline phosphate >126 U/L	15	10.0%	184	6.7%	**0.015** a
WBC >11 K/uL	60	40.0%	471	17.1%	< **0.01** a
WBC <4.5 K/uL	6	4.0%	116	4.2%	0.992
Hematocrit <34%	72	48%	857	31.11%	< **0.01** a
Platelet <150 K/uL	28	18.7%	445	16.2%	0.640
PTT >35 s	22	14.7%	426	15.5%	0.170
PT >15 s	11	7.3%	118	4.3%	0.189
INR >1.5	14	9.3%	139	5.1%	**0.027** a
*Perioperative variables:*					
Pathology type					
Aneurysm	101	67.3%	1763	64.0%	0.339
Dissection	49	32.7%	992	36.0%	
*Intraoperative variables:*					
Anesthesia time >300 min	15	10.0%	218	7.9%	0.220
Operation time >180 min	79	52.7%	827	30.0%	< **0.01** a
*Postoperative variables:*					
Pneumonia	29	19.3%	129	4.7%	< **0.01** a
Unplanned reintubation	58	38.7%	140	5.1%	< **0.01** a
Pulmonary embolism	1	0.7%	11	0.4%	0.619
Prolonged mechanical ventilation	56	37.3%	198	7.2%	< **0.01** a
Renal complications	28	18.7%	146	5.3%	< **0.01** a
Neurological complications	18	12.0%	107	3.9%	< **0.01** a
Occurrences of bleeding transfusions	100	66.7%	678	24.6%	< **0.01** a
DVT	8	5.3%	41	1.5%	< **0.01** a
Surgical site infection	2	1.3%	47	1.7%	0.708
Wound disruption	0	0%	5	0.2%	0.567
Systemic sepsis	19	12.7%	107	3.9%	< **0.01** a
URTOR	34	22.7%	215	7.8%	< **0.01** a

Abbreviations: ASA, American Society of Anesthesiologists; BMI, body mass index; CHF, congestive heart failure; CNS, central nervous system; COPD, chronic obstructive pulmonary disease; CVA, cerebral vascular accidents; DVT, deep venous thrombosis; INR, international normalized ratio; PCI, percutaneous coronary intervention; PT, prothrombin time; PTT, partial thromboplastin time; SD, standard deviation; SGOT, serum glutamic-oxaloacetic transaminase; TEVAR, thoracic endovascular aortic repair; TIA, transient ischemic attack; URTOR, unplanned return to operating room; WBC, white blood count.

a Significant *p* -values that were included in multivariate logistic regression analysis.

Following TEVAR, postoperative pneumonia, unplanned reintubation, prolonged mechanical ventilation, renal complications, neurological complications, blood transfusions, DVT, systemic sepsis, and unplanned return to odds ratio (OR) were found to be significantly associated with CEs after univariate analysis

Multivariate logistic regression analysis confirmed that ASA >3 (OR 1.71, 95% confidence interval [CI] 1.11–2.63, *p* = 0.01), emergency surgery (OR 2.80, 95% CI 1.91–4.12, *p* < 0.01), ventilator dependence (OR 2.33, 95% CI 1.30–4.17, *p* < 0.01), preoperative renal failure (OR 2.53, 95% CI 1.49–4.31, *p* < 0.01), preoperative (OR 1.84, 95% CI 1.07–3.16, *p* = 0.03), and postoperative (OR 4.02, 95% CI 2.69–6.01, *p* < 0.01) blood transfusion, WBC >11 K/uL (OR 2.45, 1.57–3.84, *p* < 0.01), postoperative prolonged mechanical ventilation (OR 1.94, 95% CI 1.17–3.24, *p* = 0.011), and unplanned reintubation (OR 5.52, 95% CI 3.46–8.82, *p* < 0.01) were significantly and independently associated with post-TEVAR CEs ( Table 2 ).

**Table 2:** Multivariable logistic regression identifying predictors of cardiac events following thoracic endovascular aortic repair

Outcome	Odds ratio	CI interval (95%)		*p* -Value
		Lower bound	Upper bound	
ASA >3	1.710	1.113	2.627	**0.014**
Emergency case	2.804	1.909	4.118	**<0.01**
Dependent functional status	1.375	0.786	2.405	0.265
Ventilator dependence	2.326	1.296	4.174	**<0.01**
Renal failure	2.534	1.491	4.308	**<0.01**
Bleeding disorder	1.514	0.973	2.356	0.066
Blood transfusion	1.840	1.073	3.155	**0.027**
Systemic sepsis	1.405	0.891	2.216	0.143
Blood urea nitrogen ≥23 mg/dL	1.006	0.594	1.705	0.981
Creatinine ≥1.2 mg/dL	1.489	0.898	2.469	0.122
Albumin <3.5 mg/dL	1.538	0.953	2.482	0.078
Total bilirubin >1.2 mg/dL	1.178	0.605	2.295	0.630
SGOT >35 U/L	1.246	0.720	2.155	0.432
Alkaline phosphate >126 U/L	1.079	0.575	2.025	0.813
WBC >11 K/uL	2.452	1.567	3.835	**<0.01**
Hematocrit <34%	1.025	0.650	1.616	0.915
INR >1.5	1.737	0.878	3.438	0.113
Operative time >180 min	0.958	0.401	2.288	0.922
Pneumonia	1.275	0.718	2.265	0.407
Unplanned reintubation	5.520	3.455	8.820	**<0.01**
Prolonged mechanical ventilation	1.943	1.166	3.240	**0.011**
Renal complications	1.564	0.875	2.796	0.131
Neurological complications	1.209	0.634	2.306	0.565
Bleeding transfusions	4.018	2.685	6.013	**<0.01**
DVT	1.733	0.693	4.334	0.240
Sepsis	0.746	0.376	1.477	0.400
URTOR	1.094	0.518	2.311	0.815

Abbreviations: ASA, American Society of Anesthesiologists; CI, confidence interval; DVT, deep venous thrombosis; INR, international normalized ratio; OR, odds ratio; SGOT, aspartate aminotransferase; TEVAR, thoracic endovascular aortic repair; URTOR, unplanned return to operating room; WBC, white blood count.

Overall, patients who experienced CEs following TEVAR were more likely to have prolonged hospitalization (13.16 ± 14.72 vs. 8.3 ± 9.32 days, *p* < 0.01) and higher readmission rates (19.3 vs. 8.2%, *p* < 0.01; Table 3 ).

**Table 3:** Associations between cardiac events and total length of hospital stay, readmission, and mortality

Variables	Cardiac events *N* = 150	% Cardiac events	No cardiac events *N* = 2755	% No cardiac events	*p* -Value
Total length of hospital stay in days (mean ± SD)	13.2 ± 14.7		8.3 ± 9.32		< **0.01**
Readmission	29	19.3%	226	8.2%	< **0.01**
Mortality	91	60.7%	151	5.5%	< **0.01**

Abbreviation: SD, standard deviation.

## Discussion

Prior studies have evaluated risk factors for CEs in patients undergoing major vascular surgery 5 6 ; however, there are scarce data that provide information on perioperative risk factors for CEs in TEVAR patients. In fact, the only available study that provides cardiac risk stratification in TEVAR patients was one by Ganapathi et al 1 which reported on only preoperative risk factors for CEs following TEVAR at a single institution. To the best of our knowledge, our study thus becomes the first multicenter study to present a perioperative risk prediction model that allows for identifying patients at highest risk of CEs following TEVAR. In evaluating risk factors for post-TEVAR CEs, it is essential to appreciate the burden that CEs place on surgeons, patients, and the health care system. In our present study, CEs were associated with high rates of mortality, readmission, and prolonged hospitalization.

Overall, this was a large ACS-NSQIP study of 2,905 patients who underwent TEVAR for descending thoracic aortic aneurysm or dissection, of whom 150 (5.2%) experienced CEs. This 5.2% incidence of CEs is comparable with published results. 1 2 Of the 150 patients who experienced CEs, 67.3% presented with descending thoracic aortic aneurysm compared with 32.7% with dissection. However, there were no significant differences for incidence of post-TEVAR CEs between aneurysm and dissection patients. This study's overall 30-day mortality rate of 9.1%, which is similar to other reported studies, 7 8 9 suggests decreased mortality compared with open surgery. In-hospital mortality for open repair of descending thoracic aortic aneurysms and/or dissections has been reported as high as 32.1%. 7 10

Our analysis suggests that ASA >3, emergency surgery, dependent functional status, preoperative renal failure, leukocytosis, unplanned reintubation, and perioperative blood transfusion were independently associated with post-TEVAR CEs. After TEVAR, unplanned reintubation was an associated significant and independent risk factor.

These findings are perhaps not surprising. Generally, patients who undergo emergency major vascular surgery usually present with life-threatening conditions 11 that make them more susceptible to develop postoperative CEs. There is also evidence that patients who are functionally dependent have a greater medical burden, both acutely and chronically. 12 Scarborough et al 12 observed that among patients who underwent complex general or vascular surgery, functional dependency was independently associated with postoperative morbidity and mortality. Crawford et al 13 also observed that preoperative functional dependence is associated with all adverse postoperative complications in patients who underwent infrainguinal surgical bypass.

Similarly, preoperative renal failure has been associated with poor outcomes following major vascular and endovascular surgery. 14 Patients in renal failure often have significant coronary artery stenosis resulting from vascular calcification and atherosclerosis, in addition to ischemia caused by decreased oxygen delivery, 15 which may make them more susceptible to developing postoperative CEs. In fact, CEs have been reported concurrently as the largest single cause of mortality in dialysis patients. 16 17 18 The finding that ASA > 3 is independently associated with CEs is expected since other reports confirm a significant association between comorbidity and adverse postoperative outcomes. 19 20 21 22 23

Findings of our study suggest that perioperative blood transfusions independently increase rates of post-TEVAR CEs; however, this association should be interpreted cautiously. In fact, even though some studies demonstrate an independent association between perioperative blood transfusion and adverse postoperative outcomes, 24 25 26 27 it is worthwhile to note that patients who are transfused perioperatively, in general, tend to be sicker and have several comorbid conditions including anemia which independently increase their risks of postoperative adverse outcomes. 28 29 30 31 Nonetheless, that PRBCs contain proinflammatory cytokines which increase systemic inflammatory response, and have been noted to adhere to vascular endothelium leading to reduced microvascular flow and tissue hypoxia 25 32 33 demonstrate that emphasis should be placed on continuous cardiac monitoring of patients who undergo perioperative blood transfusions.

The effect of mechanical ventilation on cardiovascular performance is well-reported. 34 35 Mechanical ventilation induces changes in intrapleural or intrathoracic pressure and lung volume and causes changes in atrial filling or preload, impedance to ventricular emptying or afterload, heart rate and myocardial contractility. 34 35

Unplanned reintubation is an unexpected occurrence after surgery and usually represents a deteriorating postoperative course. Patients who undergo unplanned reintubation have been reported to have higher complication rates and mortality. 36 37 38 Though reasons for unplanned reintubation for individual patients are not provided by the ACS-NSQIP database, indications usually range from emergency management of failed extubation, reintubations for emerging complications such as refractory hypotension or inability to protect airway, or emergency tracheostomy, 4 36 all of which represent clinical deterioration.

Lastly, it is worthwhile to note that the benefits of preoperative laboratory assessments in the patients undergoing surgery have been discussed. 39 40 In the present study leukocytosis was observed as an independent predictor of CEs after TEVAR. Preoperative leukocytosis may reflect an inflammatory response that might be associated with vascular injury and organ dysfunction, 39 40 increasing the likelihood of CEs.

### Limitations

The present study demonstrates that the cause of CEs following TEVAR is multifactorial. These results should be interpreted within the context of some limitations. First, the retrospective and observational nature of the study makes it difficult to accurately prove causation. Nonetheless, our multivariate analysis helped to control for multiple covariates. Second, the ACS-NSQIP database has its inherent limitations. The ACS-NSQIP only provides information on 30-day morbidity and mortality and does not elaborate on severity of some of the comorbid conditions. It is possible that some variables that were not significant in our study could be the predictors of post-TEVAR CEs in the long term. Similarly, the ACS-NSQIP database classifies cases based on CPT codes; hence, anatomical differences, procedural technique differences, and complication severity remain unclear. Additionally, though no statistical difference was observed in the incidence of post-TEVAR CEs between patients with aneurysm and dissection, the ACS-NSQIP database does not differentiate between Stanford Type A and Type B aortic dissections, making it difficult to account for any associations. In addition, the ACS-NSQIP database disproportionately represents data from large academic quality-seeking institutions due to the costs associated with participating and contributing to this database. 41 Hence, a disparity of risk factors may exist at other nonparticipating institutions, making our results nongeneralizable. Finally, ACS-NSQIP database is also subject to recording errors even though trained individuals at each institution are assigned to input data. Despite these limitations, the ACS-NSQIP database is a validated database with high inter-rater reliability that provides accurate data and allows for understanding of postoperative patient outcomes. 41 42

## Conclusions

This is the first large multicenter study that provides a practical model to predict risk factors for CEs following TEVAR. Emergency surgery, ASA >3, dependent functional status, ventilator dependence prior to surgery, perioperative blood transfusions (preoperative and postoperative), preoperative leukocytosis, preoperative renal failure, postoperative prolonged mechanical ventilation, and postoperative unplanned reintubation are strong independent predictors of CEs following TEVAR. Recognizing these identified risk factors should allow for increased preventative approaches to decrease CEs and improve TEVAR outcomes. Future studies should prospectively evaluate these predictors to comprehensively inform surgeons on risk stratification for post-TEVAR cardiac outcomes.
